# Of Sudden Deaths, in the Puerperal State, from Dynamic Lesions of the Nervous Centres

**Published:** 1857-07

**Authors:** Ch. Dubrenilh


*Of Sudden Deaths in the Puerperal State, from Dynamic Lesions of the Nervous Centres; read before the Medical Society of Bordeaux, the 19th of January,* 1857, by Dr. CH. DUBRENILH. Translated, by the Editors, from "The L'Union Medicale de la Gironde."

The study of sudden deaths in the puerperal state has not received much attention in works on obstetrics, even the most recent. With the exception of some observations, published recently, calling attention to the possibility of this fatal termination, we have but divers and contradictory opinions to explain it. I do not intend, gentlemen, to discuss, at present, but one of the points still obscure, of this great question, whose history I intend to write some time hence. The subject of the present meeting will be the dynamic lesions of the nervous centres, which may induce the sudden death of a woman who is in labor, or who has been delivered for a longer or shorter time. I will include all those cases in which death has taken place suddenly, without any cause whatever being found in the apparent state of the nervous centres or other organs to explain it.

Every thing leads us to believe, says M. Aran, in his excellent thesis for the *aggregation*, that, in some of the cases, there ought to exist lesions, which science will discover one day. But there are some in which we will be forced to admit an intimate lesion of the nervous substance, a lesion momentary and transient, whose effects may be promptly fatal, in which the cadaveric examination will not afford us any traces.

Several years ago M. Chanmel, in one of his clinical lectutes, mad the remark that, among four hundred and fifty individuals who died at Charity Hospital, he had seen seven die in a way quite unexpected, without the most careful researches affording any thing which could explain the quick and fatal termination. The subjects of these observations were all young women, but recently delivered, whose condition apparently was the most satisfactory.* When a woman has conceived, a profound modification of the entire organism takes place, which exalts her sensibility, and renders her more susceptible and more impressible to the action of physical and moral agents. It is this modification which constitutes the puerperal state, a condition produced by conception, developed by the pregnancy, augmented by the pains of labor, prolonged and debilitating during nursing, and not ceasing until the woman has resumed the habitual condition. In the production of sudden deaths in the puerperal state, the physician must never lose sight of the important rôle which the nervous system plays as an agent of transmission. If the boldest theory can not always imagine or discover a cause there, or the cadaveric examination does not afford us any trace of the lesion, physiological and anatomical investigations can, at least, aid us a great deal in its conception.

We range in the class of sudden deaths by dynamic lesions of the nervous centres, the cases in which death has taken place suddenly in consequence of syncope, of pain, and of a high moral emotion.

1st. *Syncope.*—There is nothing more frequent than syncope during the puerperal state. This morbid phenomenon is observed often during pregnancy and labor, and sometimes at a time more or less prolonged after delivery. We have seen many times feeble or nervous women fall into syncope from the most trifling cause, and sometimes without any known cause. This condition may occur often, especially in the first months, and is always very alarming to those who are present, being very often fatal, especially after delivery. In the absence of appreciable anatomical lesions, syncope is one of the causes which has been the most frequently supposed to explain the sudden death in the puerperal state.

The energy of the brain, says Cullen, being evidently, on different occasions stronger or weaker, it seems that its action can not augment without being necessarily followed by a state of debility. We may consequently understand, according to this law of nervous power, how the sudden and violent action of the energy of the brain is sometimes followed by such a diminution in the force of this energy, that it produces syncope and even death. It is in this way, I suppose, according to the same principle, that an acute pain may sometimes augment the energy of the brain to a degree more considerable than it can support, from which there results a diminution of force which must occasion fainting. But the consequence of this principle becomes more evident at the time of the syncope, which supervenes easily, when a severe pain ceases all at once. It appears that it is in a manner entirely analogous, that syncope succeeds instantly on a violent effort, continued for a long time, whether this effort is voluntary or depending on a particular disposition. It is, also, in this way that syncope attacks a woman during labor.‡

Bichat believed that syncope affects the brain secondarily, and that it

* Clinique des Hospit., † 2 p. 154.

‡ Cullen, Compend. de. Med.

is the heart whose action is first interrupted, determining by its momentary death the deficiency of action in the brain. From the different considerations in which he has viewed this subject, he concludes that the primitive source of the disease in syncope, is always in the heart; that this organ does not cease to act at that time because the brain is interrupted in its action, but that this latter dies because it does not receive from the former the fluids which habitually stimulate it, and that the usual expression *mal au cœur* expresses, with exactness, the nature of this disease.

Several modern authors agree with M. Bichat. M. Piorry believes that the circulation holds the first rank in the order of the causes of syncope, sending no longer to the brain a stimulating liquid; from this there follows a suspension of the functions of the encephalon, and, consequently, more nervous influence is sent to the heart, which arrests it and produces death. The uncertainty of the causes of sudden deaths in the puerperal state has led M. Robert to ask of the Surgical Society, "If we should not seek the disposition in that chloro-anæmic condition observed recently by M. Cazeaux in a great number of women during pregnancy?" Among the facts that this Surgeon has observed he thinks a chlorotic state existed.

The augmentation of the fluidity of the blood, which hematological researches attributed to the augmentation of the quantity of water, coinciding with a general diminution of organic materials, correspond to these morbid states designed under the name of chlorosis and anæmia. It is to M. Cazeaux, an eminent correspondent of our Society, that we owe the explanation of a great number of accidents of the puerperal state which, until very recently, were attributed to an augmentation of the coagulability of the blood or plethora.

"We have sought to verify by facts," says this distinguished academician, "the value of the indications that we have deduced from the documents furnished by the experiments of two learned professors, M. M. Gavaret and Andral, and we must say that practice confirms the theory. It is, also, with an entire confidence that we proclaim boldly, to-day, what we affirmed timidly in a simple note; the hydrœmia is, in pregnant women, the most frequent cause of functional troubles attributed, until the present time, to plethora."

It is established from the table, which M. Regnauld has given in an excellent thesis, that in pregnant women, not only the serum is found in greater abundance relative to the fibrine and globules, but that it becomes itself less rich in solid parts, which necessarily contributes to increase the total mass of water which the blood contains.

These results of chemical analysis, as also the physical qualities of the blood agree with those which we find in women in the puerperal state; on the other hand, chlorosis aud anæmia being essentially characterized by the diminution of its globules, and the augmentation of the water, it is rational to conclude, that all these functional troubles, observed during pregnancy, ought to be attributed to chloro-anæmia; they are, besides, identical with those which have been observed in chlorotic women.— These considerations, and the existence of chloro-anæmic state, are not indifferent for the subject which occupies us; for with subjects prostrated by losses of blood, or by abundant evacuations, whatever may be their nature, in chlorotic persons we have seen sudden death take place in a very unexpected manner, and in the most various circumstances. This particular condition of the blood may, in the special circumstances of the puerperal state, have an immense influence in the production of these mortal syncopes, and become the cause of sudden death. To this particular state of chloro-anæmia, in the pregnant woman, may be very rationally attributed several of these fatal terminations, in which the scalpel has failed to find any trace of appreciable lesion.

It is justly, according to M. Devergie, that this want of pathological alteration coincides always with death by syncope, which forms, so to speak, of its characters, one of its concomitant circumstances. Very often syncope will be a result entirely mechanical, an effect of the sudden withdrawal of the blood from the brain; the nervous center falls then into a nervous collapse, in consequence of which syncope, and even death may supervene. This may be the cause of syncope after accouchment. In effect, the uterus, assuming then, rapidly, dimensions smaller than those which it had during gestation leaves a free expansion, to the large vessels of the pelvis, and then a very powerful sanguine raptus takes place in this region, at the expense of the liquid which nourishes the superior extremities; this is a very different cause from that of a syncope coming on after a hemorrhage. The accoucher, says M. Cazeaux, must know, that the syncopes which come on after delivery, are not always the result of a hemorrhage; we have observed it quite often in cosequence of very rapid labors. The womb being emptied suddenly, the hypogastric vessels cease immediately to be compressed as they were during the latter months of gestation; the circulation becomes more free and easy, and the rapidity with which the blood abandons the head and inferior extremities, to flow into the vessels of the inferior abdominal regions, produces syncope very often.

*Observation 1st.* The young wife of one of our confréres was affected, during the last three months of her pregnancy, by vomiting, so stubborn that she could retain nothing. Continued fever followed, with nocturnal paroxysms, with great loss of flesh, and prostration. She completed the term of a very painful pregnancy—the labor lasted ten hours. During the stage of expulsion, which lasted four hours, an imperious duty obliged me to absent myself. Immediately after the spontaneous termination of the labor, the unfortunate woman had a first syncope; and, although the uterus properly contracted, did not allow any hemorrhage, she expired three-quarters of an hour after, in spite of the internal and external use of the most powerful tonics.—*Cazeaux*, p. 814.

This same mechanism of syncope holds good when it manifests itself during labor, and when it is followed by death. The loss of a great quantity of water has the same effect of emptying, suddenly, the uterus, and producing a syncope analogous to that which is observed in cases of abdominal paracentesis.

*Observation 2d.* A poor woman of Charity Hospital had been in labor since five o'clock. The membranes ruptured, a great quantity of water escaped, and from this time she felt excessively weak. Feeling the desire of using the night-vessel, she sat upon it, and made some expulsory efforts, and fell over in a syncope. She was immediately placed in a horizontal position; but she was scarcely laid upon the bed before she was dead. At the autopsy nothing was found to explain the death.—*Davis.*

When sudden death takes place, a longer or shorter time after delivery, the result of the experiments of MM. Piorry and Marshall Hall, on the production of syncope, is perfectly applicable. Two things may then produce it: a deficient excitation of the cerebral substance by the deviated blood, by reason of the equilibrium of weight, from its normal course, and the sitting position. Often, indeed, as the following observations prove, it is at the moment when the patients raise up, or when they are raised, that these terrible accidents take place. One of the most curious results, in regard to deaths of this kind, says M. Aran (thesis cited), is the influence of position. Some animals who appeared dead, were re-animated by placing them in a horizontal position, or by placing the head lower than the rest of the body. Some individuals, weakened by suffering, whose blood has been impoverished, faint as soon as they assume the erect position; if they are permitted to remain in the standing or sitting position they do not recover their senses, but lying down they recover immediately. This suspension of cerebral innervation may be followed by the definite suspension of the other functions—a suspension which nothing can remove. We have then the origin of the deaths so frequent in convalescence after accouchement.

*Observation 3d.* A young woman, 25 years of age, the mother of two children, being much agitated on account of political troubles, decided to go to Versailles to be delivered. She was very happily delivered; nothing unusual occurred; her health was perfectly good until the ninth day, when sitting up in bed, about to eat something, she fainted, and died immediately, in spite of the usual means. (Dehous, thesis 1854.)

*Observation 4th.* M. Robert has seen two such cases. A young woman, who was not a *primipara*, died the sixteenth day, although still in bed, while making some part of her toilet.

*Observation 5th.* Another woman, the mother of several children, died on the 16th day, when she began to take her breakfast. No autopsy was had. (*Surgical Society*, meeting 7th January, 1852.)

*Observation 6th.* An accoucheur was called to attend a young woman, pregnant with her first child, and at term. The labor had commenced, and while he withdrew for some time, a syncope came on, without any known cause. On his return this fact was communicated to him; but, as the patient appeared perfectly well, no attention was paid to it, and the delivery took place without an untoward symptom. Three days afterward, she took a purgative, and while at stool she fell over, and expired immediately. (Merriman, *Union Medicale*, 23d June, 1853.)

In a memoir, entitled "Researches on Sudden Deaths in the Puerperal State," published by Mr. McClintock, of Dublin,* we find some remarks on a morbid state, described by the name of idiopathic asphyxia, which is perhaps but a syncope. Idiopathic asphyxia, says Chrystison, causes death, almost instantaneously, in some minutes, or rather, in some cases at the end of an hour and a half. The symptoms are those of syncope. The only alteration which is found in the cadaver consists in the flaccidity of the heart, with an almost entire emptiness of its cavities. In the original memoir, which he has published, on this disease, in the first volume of the Medico-Chirurgical Transactions, M. Chevallier relates the case of the sudden death of a lady, three hours after she had been delivered of twins. He made an autopsy, and from what he observed he was

* Union Medicale, 1855, p. 294.

led to conclude, that death could only be attributed to this particular form of asphyxia.

The same author reports, from Morgagni, a case of rapid death in the puerperal state, which seemed to be owing to the same cause. M. Backer gives an account of two cases, which support the preceding. In these two cases, death supervened quite suddenly, when it was least expected, a few days after delivery. At the autopsy no other condition could be found to explain the death, than an abnormal flaccidity of the heart, with complete absence of blood in its cavities. If we admit this cause of death, adds McClintock, we do not see why it may not attack puerperal women. Much more, if we admit, as certain persons do, and as I am not far from believing, that idiopathic asphyxia is nothing else than a variety or form of syncope, we must be still more disposed to admit the possibility of its production in women in the puerperal state; that is to say, in a state in which their constitution is already weakened by the parturient act, and which has for one of its chief characteristics, an abnormal disposition to morbid action, a particular excitability of the vascular system, and a pathological susceptibility of the nervous system. It requires several days for women to recover from the shock produced by labor, and during this period, whose duration varies, necessarily, in different circumstances, the vital resistance is diminished. Consequently, all kinds of impressions, which affect the body or mind, surprise the economy in much less favorable conditions. For all these reasons, it seems to me impossible not to admit, that several cases of sudden death, in the puerperal state, still unexplained, must be attributed to idiopathic asphyxia, or to a syncope.—*Cincinnati Medical Observer.*

[TO BE CONTINUED.]

